# Thyroid hormones in diabetes, cancer, and aging

**DOI:** 10.1111/acel.13260

**Published:** 2020-10-13

**Authors:** Benoit R. Gauthier, Alejandro Sola‐García, María Ángeles Cáliz‐Molina, Petra Isabel Lorenzo, Nadia Cobo‐Vuilleumier, Vivian Capilla‐González, Alejandro Martin‐Montalvo

**Affiliations:** ^1^ Department of Cell Therapy and Regeneration Andalusian Center for Molecular Biology and Regenerative Medicine‐CABIMER Junta de Andalucía‐University of Pablo de Olavide‐University of Seville‐CSIC Seville Spain; ^2^ Biomedical Research Network on Diabetes and Related Metabolic Diseases‐CIBERDEM Instituto de Salud Carlos III Madrid Spain

**Keywords:** cancer, diabetes, health span, hyperthyroidism, hypothyroidism, life span, thyroid hormones

## Abstract

Thyroid function is central in the control of physiological and pathophysiological processes. Studies in animal models and human research have determined that thyroid hormones modulate cellular processes relevant for aging and for the majority of age‐related diseases. While several studies have associated mild reductions on thyroid hormone function with exceptional longevity in animals and humans, alterations in thyroid hormones are serious medical conditions associated with unhealthy aging and premature death. Moreover, both hyperthyroidism and hypothyroidism have been associated with the development of certain types of diabetes and cancers, indicating a great complexity of the molecular mechanisms controlled by thyroid hormones. In this review, we describe the latest findings in thyroid hormone research in the field of aging, diabetes, and cancer, with a special focus on hepatocellular carcinomas. While aging studies indicate that the direct modulation of thyroid hormones is not a viable strategy to promote healthy aging or longevity and the development of thyromimetics is challenging due to inefficacy and potential toxicity, we argue that interventions based on the use of modulators of thyroid hormone function might provide therapeutic benefit in certain types of diabetes and cancers.

## Graphical abstract

Thyroid hormones are central in the control of health and disease. Here we review the latest findings in thyroid hormone research in the field of diabetes, cancer, and aging. Aging studies indicate that the direct modulation of thyroid hormones is not an viable strategy to promote healthy aging. However, although challenging, interventions based on the use of modulators of thyroid hormone function might provide therapeutic benefit in certain types of diabetes and cancers.

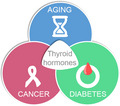

## 1 INTRODUCTION

**FIGURE 1:**
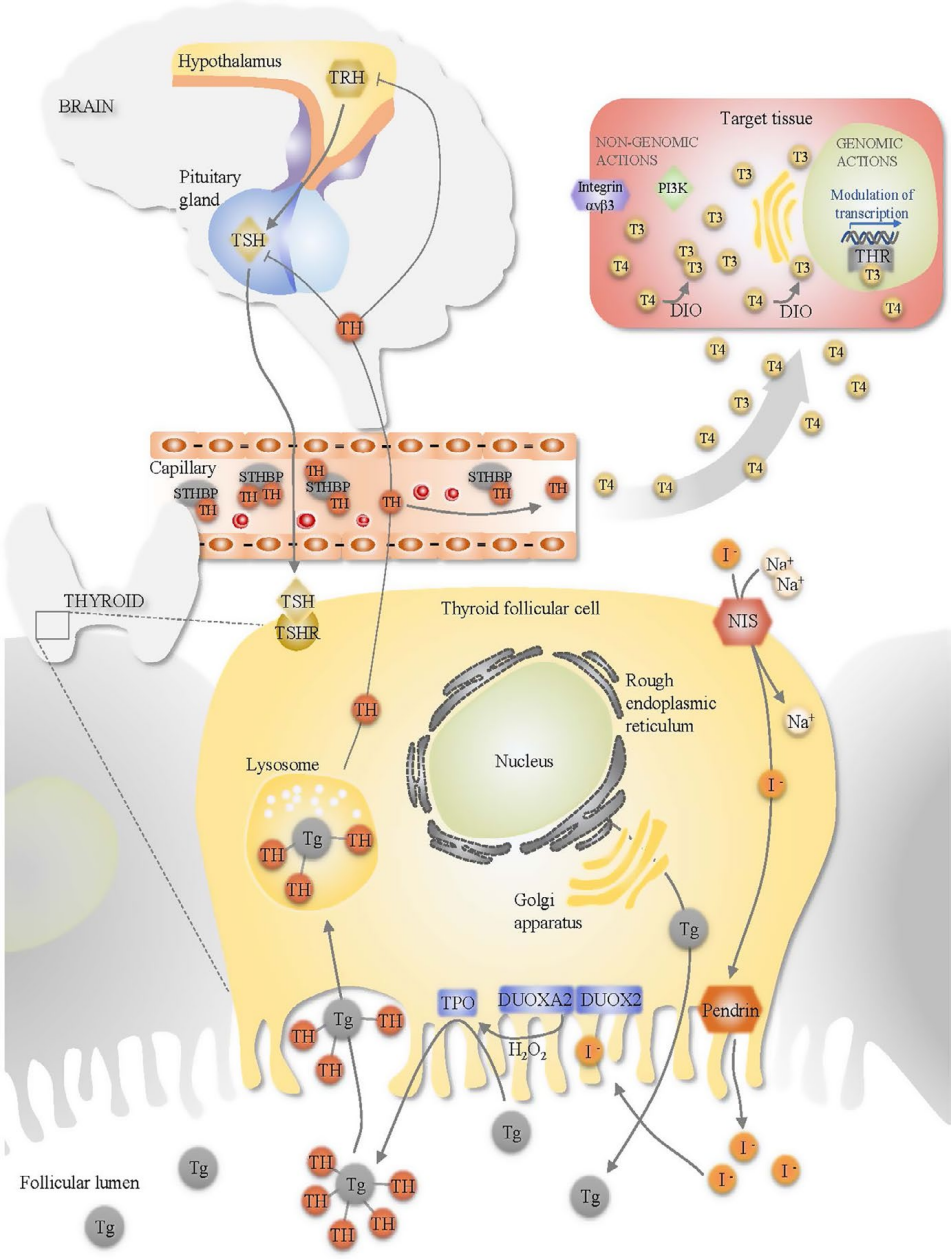
Scheme summarizing TH synthesis. TRH is produced by a specific group of neurons located in the paraventricular nucleus of the hypothalamus. When TRH reaches the pituitary gland, it binds to the TRH receptor expressed in the thyrotrophs, stimulating the expression and secretion of TSH into the circulation. TSH reaches the thyroid gland and binds to the TSHR located in the cell membrane of the thyroid follicles, setting in motion TH production in a process that includes the induction of iodine uptake by the NIS. Iodide is mobilized to the colloid via Pendrin action, and it is then oxidized by the thyroid peroxidase (TPO) using H_2_O_2_. Iodination of tyrosine residues forms monoiodotyrosines and diiodotyrosines that are coupled to form T3 and T4. T3 and T4 bound to TG are released in the colloid of the follicle. When needed, iodinated TG is catabolized in thyroid follicular epithelial cells to produce T3 and T4, which are then released into the circulation. In the bloodstream, THs can be found either free or bound to serum TH‐binding proteins (STHBP), such as thyroxine‐binding protein, transthyretin, and albumin. Free THs are able to enter into target cells in target tissues via membrane transporters. In target cells, deiodinases generate T3 from T4 by removing the iodine located at the 5´ position of T4. Intracellular T3 acts via genomic actions binding to the THR, where modulate gene expression, or via non‐genomic actions affecting signaling pathways such as integrin αvβ3 and PI3 K. THs also act via the nuclear THRβ in the hypothalamus and the pituitary to inhibit TRH and TSH production and secretion, completing a negative feedback loop that maintains physiological levels of THs. DIO: deiodinase. DUOX2: dual oxidase 2. DUOXA2: dual oxidase maturation factor 2. I^−^: iodide. STHBP: serum TH‐binding proteins. Na^+^: sodium. NIS: sodium‐iodide symporter. T3: triiodothyronine. T4: thyroxine. Tg: thyroglobulin. TH: thyroid hormone. THR: thyroid hormone receptors. TPO: thyroid peroxidase. TRH: thyrotropin‐releasing hormone. TSH: thyrotropin. TSHR: thyrotropin receptor

Thyroid hormone (TH) production is a tightly regulated process controlled by a classic negative feedback loop involving the hypothalamus, the pituitary, and the thyroid, which has led to the common name hypothalamus–pituitary–thyroid axis (Figure 1). The thyrotropin‐releasing hormone (TRH) is produced in the hypothalamus. Once released, TRH reaches the pituitary gland and binds to the TRH receptor and stimulates the production and secretion of thyroid‐stimulating hormone (TSH), also known as thyrotropin (Liu et al., 2019). In the thyroid, TSH binds to the TSH receptor (TSHR) and induces TH production. When needed, triiodothyronine (T3) and tetraiodothyronine (T4), also known as thyroxine, are released into the circulation. In the hypothalamus and the pituitary, THs act *via* the nuclear TH receptor β (THRβ) to inhibit TRH and TSH production and secretion, completing a negative feedback loop that maintains the physiological levels of TRH, TSH, and THs.

In target cells, deiodinases (DIO2 and DIO3) generate T3 from T4 by removing the iodine located at the 5′ position of T4. The expression of the different deiodinases is cell‐type and tissue‐specific, which provides a mechanism to control TH actions irrespective of circulating TH levels (Gereben et al., 2008; Schweizer et al., 2008). Intracellular T3 acts via binding to the TH receptor α (THRα) and THRβ, which display high affinity for DNA sequences called TH response elements (TREs). Upon ligand binding, THRs assemble into a co‐activator complex with histone acetyltransferase activity that is recruited to stimulate transcription (Lonard & O'Malley, 2007; Perissi et al., 2010). Moreover, THR interacts with other nuclear hormone receptors, such as peroxisome proliferator‐activated receptors, retinoid X receptors, retinoic acid receptors, and liver X receptors that allow binding to a wide repertoire of nucleotide sequences that contribute to regulate different metabolic pathways, including cholesterol, glucose, and fatty acid metabolism in different tissues (Brent, 2012; Kouidhi & Clerget‐Froidevaux, 2018). In addition, THs also modulate molecular pathways via protein–protein interactions such as PI3 K‐AKT‐FOXO1 and mTOR‐p70S6 K signaling, which further modulate transcription (Cao et al., 2005; Davis et al., 2016; Flamant et al., 2017; Mullur et al., 2014). In the presence or absence of THs, THRs modulate the expression of more than 80 genes, mainly involved in mitochondrial biogenesis, oxidative phosphorylation, tricarboxylic acid cycle, de novo lipogenesis, and fatty acid catabolism (Flores‐Morales et al., 2002; Jackson‐Hayes et al., 2003; Singh et al., 2018). Overall, THs enhance oxygen consumption and ATP hydrolysis and reduce the coupled state of the mitochondria inducing the catabolism of all types of energy sources (Johannsen et al., 2012; Weinstein et al., 1991). At the organismic level, THs increase the basal metabolic rate, which is defined as rate of energy expenditure per time at rest.

**FIGURE 2:**
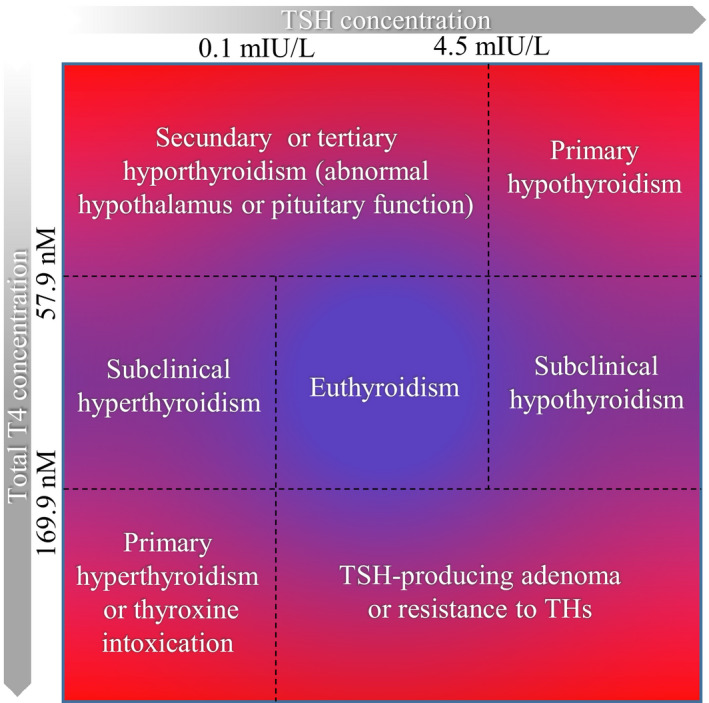
Reference ranges and medical conditions associated with thyroid dysfunction. This figure defines reference ranges for primary hypothyroidism, secondary hypothyroidism, tertiary hypothyroidism, subclinical hypothyroidism, euthyroidism, subclinical hyperthyroidism, primary hyperthyroidism/thyroxine intoxication, and TSH‐producing adenoma/resistance to THs. TSH and T4 levels were defined as Hollowell et al. Total thyroxine can be converted from nM to µg/dl, dividing by 12.87. THs: thyroid hormones. TSH: thyrotropin

THs are required for the development and maturation of several tissues and general well‐being (Ng et al., 2013; Nunez et al., 2008). Hollowell et al. have defined the normal reference ranges of total T4 at 57.9–169.9 nM and TSH at 0.39–4.6 mIU/L (Figure 2) (Hollowell et al., 2002). It is estimated that in the general population the prevalence of TH alterations is ~0.5%–4% in areas with sufficient iodine exposure. There are different types of TH alterations (hyperthyroidism, subclinical hyperthyroidism, subclinical hypothyroidism, and hypothyroidism) that lead to different clinical symptoms (Figure 2) (Hollowell et al., 2002). Recent epidemiological meta‐analyses have determined a clear association of TH alterations with mortality risk in the general population (Brandt et al., 2011; Kovar et al., 2015; Thvilum et al., 2012).

Clinical hypothyroidism, also known as overt hypothyroidism, is associated with metabolic deregulations, such as hypercholesterolemia, and increased low‐density lipoprotein (LDL) levels, that increase the risk of developing diabetes mellitus (DM) and cardiovascular complications (Cappola & Ladenson, 2003; Duntas, 2002; Gao et al., 2013; Sawin et al., 1985; Taylor et al., 2013; Wang et al., 2012). Subclinical hypothyroidism has also been associated with serious complications such as improper neurocognitive health, unbalanced bone metabolism, propensity to develop type 2 DM (T2DM), cardiovascular‐associated risk factors, such as high LDL and very‐low‐density lipoprotein (VLDL) levels, hypertriglyceridemia, hypertension, atrial fibrillation, and obesity, as well as low levels of high‐density lipoprotein (HDL) and premature mortality (Auer et al., 2001; Biondi & Cooper, 2008; Biondi et al., 2002; Ceresini et al., 2013; Han et al., 2015; Pearce, 2012; Taylor et al., 2013). On the other side of the spectrum, hyperthyroid individuals also have increased risk of developing DM and cardiovascular complications that can cause premature death (Brandt et al., 2013; Franklyn et al., 2005). However, cardiovascular complications are not associated with hypercholesterolemia in hyperthyroid patients, and they normally exhibit reduced circulating cholesterol levels (Kim et al., 2020). In the case of subclinical hyperthyroidism with severely decreased TSH, clinical data indicate an increased incidence of dementia and neurocognitive dysfunction (Aubert et al., 2017; Bensenor et al., 2010). Overt hyperthyroidism and even subclinical hyperthyroidism increase the risk of bone fractures (Blum et al., 2015; Nicholls et al., 2012; Vestergaard & Mosekilde, 2003). Altogether, clinical data clearly indicate that in the general population TH alterations are associated with poor quality of life.

### 1.1 Thyroid hormones in aging

#### 1.1.1 The regulation of thyroid hormones in aging and their role in longevity

Early observations have established that restricted thyroid function is associated with longer life span in small and large mammals, including humans (Bowers et al., 2013; Buffenstein & Pinto, 2009; Jansen, et al., 2015). Accordingly, higher serum TSH levels and/or low serum free T4 levels have been associated with longer life expectancy, indicating an important role of THs in aging (Beld et al., 2005; Gussekloo et al., 2004, 2006; Rozing, et al., 2010). Although aging may not be considered a disease, it can be argued that the aging process is not a homogeneous physiological process. From an evolutionary point of view, natural selection optimizes mechanisms and processes that allow functionality and survival until the end of the reproductive life. However, at post‐reproductive age in the majority of species, there is little natural selection to fight against mechanisms that produce unhealthy aging and death. Notwithstanding, in certain species such as humans, where familial and social interactions can be relevant for the survival of young individuals, natural selection might still have a significant role to promote longevity (Tully & Lambert, 2011). Given these interesting facts, it is difficult to define whether changes that occur in thyroid function in aged individuals are adaptive or maladaptive.

Although T4 secretion has been shown to be slightly reduced in aged individuals, the capacity to metabolize T4 by deiodination mediated by DIO1 and DIO2 is decreased in certain tissues of aged individuals, and healthy aged individuals exhibit normal T4 levels (Duntas, 2018; Mazzoccoli et al., 2010; Michalakis et al., 2013). In this line, old rats exhibiting comparable TSH levels to young ones have lower DIO1 activity and separated studies in rodents have demonstrated that hepatic levels of the TH transporter MCT8 are reduced in aged individuals, indicating that TH responsiveness changes with advancing age (Chaker et al., 2018; Donda & Lemarchand‐Beraud, 1989). Moreover, there are evidences indicating that THs might have reduced capacity to activate certain post‐receptor mechanisms of thyroid function in aged individuals (Morley, 2003).

The incidence of both hyperthyroidism and hypothyroidism in the older population has continuously risen in the past decades (Boelaert, 2013; Duntas, 2018; Surks & Hollowell, 2007). The diagnosis of hypothyroidism in elderlies is difficult since older people exhibit milder and fewer symptoms of TH deregulation. Moreover, symptoms experienced in these patients are readily interpreted as signs of the aging process, such as fatigue and neurological disorders, which difficult the diagnosis of these alterations (Martinez‐Iglesias, Garcia‐Siva, Regadera, et al., 2009). Prevalence of subclinical hypothyroidism affects ~6% of the population aged between 70 and 79 years, rising up to 10% in individuals aged over 80 years (Boelaert, 2013; Simonsick et al., 2009). Noteworthy, despite the association of subclinical hypothyroidism with several serious diseases and overall mortality in the general population, a study focused on older individuals with subclinical hypothyroidism has refuted these associations (Simonsick et al., 2009). Actually, epidemiological studies in humans have even associated subclinical hypothyroidism with a reduced risk of all‐cause mortality in individuals older than 65 years of age (Selmer et al., 2014). Furthermore, several reports have indicated that old individuals, of at least 70 years of age diagnosed with subclinical hypothyroidism (TSH levels in the range of 4.5–10.0 mIU/L), might even have certain physical function advantages and lower mortality when compared to individuals with normal thyroid function (Simonsick et al., 2009, 2016). Supporting the pro‐longevity benefits of reduced thyroid function in the elderly, thyroxine replacement therapy was not efficient in improving cognitive function in old patients with subclinical hypothyroidism (Park et al., 2010).

The prevalence of hyperthyroidism in individuals older than 60 years ranges from 1% to 15% (Bannister & Barnes, 1989; Chiovato et al., 1997; Samuels & Feingold, 2000), being autoimmune Graves’ disease is the most common etiology of hyperthyroidism in aged individuals. The majority of prospective studies associate higher TH concentrations with increased frailty and decreased functional capacity in old patients (Ceresini et al., 2011; Chaker et al., 2018; Simonsick et al., 2009). In this line, low levels of TSH or elevated levels of free T4 have been linked to compromised quality of life and increased mortality risk in the aged population (Gussekloo et al., 2004, 2006; Kramer et al., 2009; Parle et al., 2001; Singer, 2006; Waring, et al., 2012). The pro‐aging effects of THs have been studied and active T3 binding to the THRβ isoform is known to produce DNA damage and premature senescence, making a causal connection with molecular processes of accelerated aging (Zambrano et al., 2014). Moreover, the detrimental effects of chronic hyperthyroidism in life expectancy have been observed in mice and rats (Lopez‐Noriega et al., 2019; Ooka & Shinkai, 1986). In this sense, wild‐type mice exposed to T4 leading to a twofold increase in circulating T4 levels exhibit an ~50% reduction in mean and maximal life span (Lopez‐Noriega et al., 2019).

Several studies have indicated that different TH levels within the normal range could also have physiological relevance. In this regard, high–normal free T4 levels are considered a risk factor for poor outcomes for cardiovascular disease and mortality (Hogervorst et al., 2008; Simonsick et al., 2016). Several reports have determined the effect of variations of TH levels within euthyroidism on gait speed in older people, indicating that higher TH levels are associated with slower gait speed (Bano, et al., 2016; Chaker et al., 2018; Simonsick et al., 2016). Remarkably, data in the literature indicate that subjects with low–normal free T4 levels or with high–normal TSH levels were expected to live up to 3.7 years longer than individuals with high–normal free T4 levels or low–normal TSH levels (Bano et al., 2019; Chaker et al., 2018). Moreover, lower free T4 levels have been associated with better functional mobility and fitness in healthy euthyroid individuals with ages ranging from 68 to 97 years, which has led to propose low–normal free T4 levels as a marker for healthy aging (Rozing et al., 2010; Rozing et al., 2010; Simonsick et al., 2016). In this line, lower metabolic cost of walking in adults is associated with greater gait speed and a slower decline (Schrack et al., 2012, 2016). Overall, these data are in agreement with the rate of living theory of aging, suggesting that a lower metabolic demand predisposes to longer health span and life span.

### 1.2 The special case of centenarians

There is strong evidence indicating that a genetic component predisposes to longevity, which is supported by studies indicating that long‐lived parents have long‐lived offspring (Gudmundsson et al., 2000; Kerber et al., 2001; Rozing, et al., 2010). Individuals with exceptional long longevity in the Ashkenazi Jewish population and their families have been studied to determine factors that could be associated with this trait. Several reports have indicated that the offspring of Ashkenazi Jewish centenarians have higher TSH levels than the offspring of non‐centenarians and, even nonagenarians and their offspring, have increased TSH levels and/or decreased circulating T3/free T4 levels than their partners (Atzmon et al., 2009; Jansen, et al., 2015). The Leiden longevity study has also supported the association between low thyroid function with lower risk of death from cardiovascular disease and longer life spans (Rozing et al., 2010; Rozing et al., 2010; Westendorp et al., 2009). In this line, nonagenarians with the lowest family mortality history score had the highest TSH levels and slightly lower levels of free T4 and free T3 (Rozing et al., 2010). Interestingly, TSH levels were found to be higher and free T3 levels were slightly lower when the offspring was compared to their partners (Rozing et al., 2010). The Leiden longevity study also analyzed nonagenarians with at least one nonagenarian sibling, their offspring, and their partners (Westendorp et al., 2009). Remarkably, authors determined that offsprings of nonagenarian siblings had lower mortality rate, and lower propensity to develop cardiovascular disease and DM than their partners. These observations have led to the conclusion that increased TSH levels are associated with exceptional longevity, which has been further supported by studies in the oldest individuals of the general population demonstrating the association of higher TSH levels with reduced old age mortality (Atzmon et al., 2009; Gussekloo et al., 2004). An elegant work by Jansen et al. has determined TH levels and TSH secretion over 24 hours in the offspring from long‐lived families and their partners, since these hormones are known to have intra‐day oscillations due to circadian rhythms. The objective was to evaluate alterations in energy metabolism. Results obtained led to the conclusion that familial longevity is characterized by higher TSH secretion, in the absence of alterations on TH levels or energy metabolism (Jansen et al., 2015). The lack of differences in TH levels and energy metabolism is in sharp contrast with several theories of aging postulating that reduced energy metabolism promotes longer life expectancy.

Interestingly despite the fact that centenarians and their offspring tend to have lower TH function, the offspring of centenarians has a significantly lower body mass index when compared to the normal population and reduced risk of age‐related diseases, further indicating a genetic component of longevity (Terry et al., 2003). Further investigations in the Ashkenazi Jewish population have also determined that a genetic background might be responsible for increased life span, since two specific single nucleotide polymorphisms in the *TSHR* gene (rs12050077 and rs10149689) were linked to higher TSH levels in centenarians and the offspring of centenarians of this population (Atzmon et al., 2009). Based on the Leiden study and others, one can assume that in the general population of the oldest old, high levels of TSH usually are associated with healthy aging (Gussekloo et al., 2004). The fact that TSH levels and not only TH levels are associated with extended survival suggests that modulations in the negative feedback loop controlling TH production might contribute to this phenotype (Atzmon et al., 2009).

### 1.3 The molecular mechanisms of exceptional longevity

An intense area of research has been focused to identify the genetic predisposition to maximal longevity in animal models. Mutations in the *DAF2* gene, homolog of the insulin‐like growth factor 1 receptor (IGF‐1R) in *Caenorhabditis elegans* and in the IGF‐1R in *Drosophila melanogaster*, are known to extend life span (Arantes‐Oliveira et al., 2003; Tatar et al., 2001). Remarkably, the longest living laboratory mice exhibit severely reduced thyroid function as observed in the Laron (growth hormone receptor knockout), Ames (*Prop1*‐mutated), and Snell (*Pit1*‐mutated) dwarf mice (Table 1) (Brown‐Borg 2007, 2009; Brown‐Borg et al., 1996). These murine models show a healthy aging phenotype that includes, besides the restriction on thyroid function, the preservation of neurocognitive and muscular function, lower incidence of cancers, enhanced insulin responsiveness, and improved glucose tolerance (Brown‐Borg, 2009; Brown‐Borg et al., 1996; Ikeno et al., 2003; Wiesenborn et al., 2014). At a molecular level, these mice exhibit reduced signaling through the insulin and IGF‐1 pathways, which leads to restricted phosphorylation of downstream targets such as the serum/glucocorticoid‐regulated kinase and AKT. Restricted activity of these kinases promotes the translocation of FOXO transcription factors into the nucleus, where it modulates the transcription of genes that promote longevity (Brown‐Borg et al., 1996; Russell & Kahn, 2007). However, these beneficial effects on health span and/or life span might rely specifically on growth hormone production and/or sensitivity. In this context, we recently determined the direct effect of TH modulation in health span and longevity using the PAX8 knockout murine model and wild‐type mice treated or not with T4 (Lopez‐Noriega et al., 2019). PAX8 is the master transcriptional regulator of thyroid organogenesis required for TH production (Mansouri et al., 1998). Using these mice, we determined the effects in health status and life expectancy in mice suffering severe hypothyroidism, mild hypothyroidism, and severe hyperthyroidism compared with control healthy mice. T4‐treated hyperthyroid mice exhibited reduced body weight, increased food intake, and short life expectancy, indicating that elevated TH levels result in life‐threatening toxicity. Not surprisingly, the complete lack of TH production resulted in perinatal mortality (Lopez‐Noriega et al., 2019). The direct modulation of TH levels using PAX8 heterozygous knockout mice, which suffer a mild hypothyroidism due to a direct defect in the thyroid gland, while exhibiting normal circulating levels of α‐GSU of pituitary hormones in adulthood, did not result in improved health span or longer life span. As opposed to other experimental models of hypothyroidism (Hine et al., 2017; Umezu et al., 2020), we found that the PAX8 heterozygous mice faithfully recapitulate the phenotype of humans with hypothyroidism, including insulin resistance, increased white adipose tissue (WAT) mass, and increased triglyceride content in skeletal muscle and liver (Lopez‐Noriega et al., 2019). Similar to humans, these mice also exhibit reduced basal metabolic rate and obesity while maintaining normal energy intake. Moreover, PAX8 heterozygous mice exhibit poor performance in functional physical tests and accumulated oxidative damage, indicating that even mild alterations on TH levels (mild hypothyroidism) have profound effects in health span. These results indicate that low TH levels in exceptional long‐living dwarf mice are not responsible *per se* of longevity benefits. Our data support the notion that humans with exceptional longevity must have a specific genetic and/or epigenetic signature required to achieve longevity benefits (Bowers et al., 2013; Gesing et al., 2012; Jansen, et al., 2015). In addition, our data also indicate that a delicate control of TH levels and function is required to sustain health and survival and that interventions based on the modulation of THs should not be targeted to improve the quality of life or life expectancy in healthy individuals.

**TABLE 1:** Genetic alterations causing thyroid dysfunction associated with aging, DM, or cancer in mice and humans. ND: not determined

Gene	Function	Alteration	Phenotype			References
			Aging	DM	Cancer	
*DIO2*	TH activation/inactivation	Homozygous knockout mouse	ND	Several hallmarks of T2DM	ND	Marsili et al. (2011)
		SNPs in humans	ND	Several hallmarks of T2DM	ND	Canani et al. (2005); Dora et al. (2010); Mentuccia et al. (2002)
*DUOX2*	TH production	SNPs in humans	ND	ND	Predisposes to thyroid cancer	Bann et al. (2019)
*TG*	TH transport	SNPs in humans	ND	ND	Found in thyroid cancer	Hishinuma et al., 2005)
*THRα*	TH signaling	Homozygous knockout mouse	ND	Protected from hallmarks of T2DM	ND	Jornayvaz et al. (2012)
		Truncations and SNPs in cancer tissue in humans	ND	ND	Found in several types of cancer	Kim and Cheng (1830); Lin et al. (1999); Kamiya et al. (2002); Puzianowska‐Kuznicka et al. (2002); Chan and Privalsky (2006); Rosen and Privalsky (2011) McCabe et al. (1999); Cheng (2003)
*THRβ*	TH signaling	Truncations and SNPs in cancer tissue in humans	ND	ND	Found in several types of cancer	Kim and Cheng (1830); Lin et al. (1999); Kamiya et al. (2002); Puzianowska‐Kuznicka et al. (2002); Chan and Privalsky (2006); Rosen and Privalsky (2011); Cheng (2003)
*TSHR*	TSH signaling	Homozygous knockout mouse	Premature death	Glucose intolerance	ND	Abe et al. (2003); Yang et al. (2019)
		Truncations and SNPs in cancer tissue in humans	ND	ND	Mutations found in HCC and thyroid cancer	Shih et al. (2018); Russo et al. (1995); Camacho et al. (2000)
*PAX8*	Thyroid development and function.	Homozygous knockout mouse	Premature death	No	No	Lopez‐Noriega et al. (2019); Mansouri et al. (1998)
		Heterozygous knockout mouse	Unhealthy aging; normal life span	Several hallmarks of T2DM	Liver cancer	Lopez‐Noriega et al. (2019)
		Human SNPs	ND	GDM	Propensity to HCC	Martin‐Montalvo et al. (2019); Ma et al. (2017)
*PIT‐1*	TRH/TSH production	Homozygous loss‐of‐function point mutation in mouse	Delayed aging	Increased insulin sensitivity	Reduced occurrence of spontaneous cancer	Brown‐Borg (2007); Flurkey et al. (2001); Alderman et al. (2009)
*PROP‐1*	TRH/TSH production	Homozygous loss‐of‐function point mutation in mouse	Delayed aging	Increased insulin sensitivity	Delayed spontaneous occurrence of cancer	Brown‐Borg (2007) Brown‐Borg et al. (1996); Ikeno et al. (2003)

### 1.4 The effect of nutritional and pro‐longevity interventions in thyroid function

Several animals, including humans under calorie restriction, a variety of nutritional interventions that extend health span and lifespan, have low T3 and/or high TSH levels in the blood (De Andrade et al., 2015; Fontana et al., 2006; Muller et al., 2015; Ravussin et al., 2015). Remarkably, a recent report evaluating the effects of 4‐week alternate day fasting has clearly demonstrated a reduction in T3 levels in individuals adhered to the intervention, which is accompanied by improvements in markers of cardiovascular health (Stekovic et al., 2020). In this line, a caloric restriction mimetic, resveratrol, rises TSH levels and has profound effects in the thyroid gland, decreasing sodium‐iodide symporter (NIS) and thyroglobulin (TG) expression (Giuliani et al., 2017). Calorie restriction produces effects at all compartments of the HPT axis, as well as in TH target tissues. In particular, reduced hypothalamic TRH expression, reduced pituitary TSHβ expression, reduced expression of TG and secretion of T3 and T4 in the thyroid gland, and reduced hepatic DIO1 expression have been described upon different forms of caloric restriction (Boelen et al., 2008; De Andrade et al., 2015; Palkowska‐Gozdzik et al., 2017). Different nutritional interventions are also known to alter TH levels. In this regard, adult dogs consuming a low‐carbohydrate high‐protein high‐fat diet exhibited greater circulating T4 levels than dogs fed with a high‐carbohydrate low‐protein low‐fat diet (Chiofalo et al., 2019). Interestingly, Carew et al. evaluated the effect of individual essential amino acid restriction on plasma TH concentrations in chickens (Carew et al., 1997). Results indicated that changes in circulating levels of T3 under protein deficiency may be a consequence of selected amino acid deficits, since only isoleucine deficiency resulted in an elevation in plasma T3, while restrictions on other essential amino acids did not alter T3 levels when compared to control fed chickens. However, the molecular mechanisms producing these changes remain unknown.

### 1.5 Thyroid hormones in diabetes mellitus

#### 1.5.1 The implications of thyroid hormones in glucose and lipid metabolism

**FIGURE 3:**
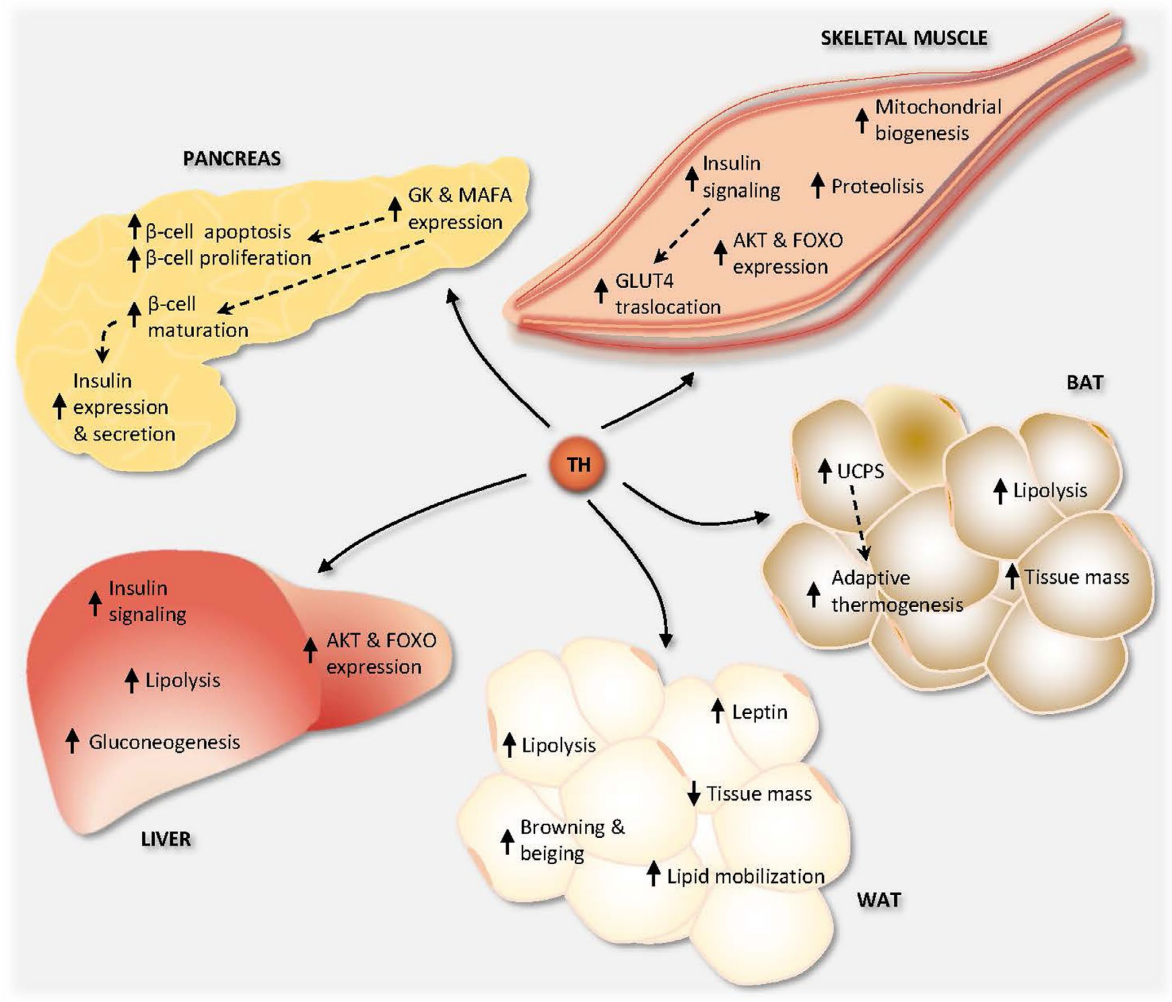
Scheme summarizing the processes regulated by THs in the main metabolic tissues. THs exert profound effects in metabolic tissues. THs enhance GK and MAFA expression in the pancreas favoring a rapid maturation and turnover of β cells. THs also potentiate insulin expression and secretion in the endocrine pancreas. Insulin‐target tissues respond increasing the activity of insulin signaling, which produces increased rates of lipolysis and gluconeogenesis in the liver and proteolysis and mitochondrial biogenesis in the skeletal muscle. Adipose tissues respond to THs increasing lipolysis and lipid mobilization. Browning/beiging of adipocytes occurs in the WAT and increasing thermogenesis via increased UCP expression and subsequent lipolysis occurs in the BAT. AKT, protein kinase B. FOXO: forkhead box O 1. GK: glucokinase. GLUT4: glucose transporter 4. MAFA: MAF bZIP transcription factor A

As previously mentioned, THs enhance oxygen consumption, inducing the catabolism of all types of energy sources (Johannsen et al., 2012; Weinstein et al., 1991). THs are efficient modulators of lipid and glucose metabolism. In particular, THs reduce circulating triglycerides and cholesterol‐containing lipoproteins. THs stimulate the expression of the Sterol response element‐binding protein 2 (Srebp‐2) (Mullur et al., 2014). Increased levels of Srebp‐2 contribute to enhance LDL receptor expression, which potentiates hepatic cholesterol uptake. Moreover, THs are known to increase simultaneously lipolysis and liponeogenesis. Actually, THs are known to increase the expression of carnitine palmitoyltransferase Iα (mitochondrial fatty acid uptake) and the acetyl‐coenzyme A carboxylase (lipogenic) (Mullur et al., 2014). A comprehensive analysis of these processes has indicated that liponeogenesis is enhanced to maintain lipid levels under conditions of high lipolysis (Oppenheimer et al., 1991). Under these circumstances, lipolysis is enhanced to provide substrates for thermogenesis. Carbohydrate metabolism is also influenced by TH. Gluconeogenesis and glycogenolysis are known to be enhanced by THs in a process that supports tissues with fuel to maintain their energy requirements. In this sense, hepatic insulin resistance in hyperthyroid individuals has been shown to increase gluconeogenesis and subsequent hepatic glucose output (Figure 3) (Klieverik et al., 2008; Potenza et al., 2009). Increased rates of gluconeogenesis are supported by increased Cory cycle activity, which implicates muscle tissue in the provision of substrates for hepatic gluconeogenesis (lactate and certain amino acids such as alanine and glutamine). This process represents a dynamic buffer of glucose that allows its use by other tissues under glucose requirements when needed. Within the liver, THs are known to enhance the expression of the phosphoenolpyruvate carboxykinase, the rate‐limiting step in gluconeogenesis, supporting a direct role of THs in the regulation of these processes (Park et al., 1999). Studies in mice exposed to T4 mimicking hyperthyroidism have also indicated that insulin signaling is active in insulin‐target tissues even under fasting conditions, due to a deregulated function of the endocrine pancreas (e.g., increased insulin secretion and subsequent levels in circulation) (Lopez‐Noriega et al., 2017). Overall, compelling data in the literature indicate that THs produce effects in several, if not all, tissues involved in glucose and lipid homeostasis (Figure 3).

#### 1.5.2 Alterations of thyroid hormones in diabetes mellitus

The relationship between alterations on thyroid function and the development of different types of DM has been the focus of intense research. The prevalence of hyperthyroidism in subjects suffering DM is greater than in non‐diabetic subjects (Biondi et al., 2019), and a nationwide Danish study has determined that patients suffering hyperthyroidism have greater risk to develop DM (Brandt et al., 2013). Among adult patients with T2DM, ~4.4% have overt hyperthyroidism and 2%–4% have subclinical hyperthyroidism (Biondi et al., 2019). Interestingly, improved diabetic control in T2DM patients normalizes TSH levels in patients with subclinical hyperthyroidism, suggesting that treatments improving T2DM might contribute to normalize thyroid function (Celani et al., 1994). However, a recent report has indicated that non‐diabetic patients diagnosed with hyperthyroidism have increased risk to develop T2DM later in life, suggesting that thyroid dysfunction might precede diabetogenic processes (Chen et al., 2019). In this line, while hyperthyroid patients exhibit increased basal hepatic glucose production and increased fasting insulin levels when compared to healthy individuals, hyperthyroid patients treated with methimazole rendered euthyroid, exhibited significantly reduced levels on the same parameters, reaching the levels of the healthy control group (Cavallo‐Perin et al., 1988). An independent report has also indicated that patients with overt or subclinical hyperthyroidism exposed to a glucose tolerance test have higher circulating levels of glucose and insulin (Maratou et al., 2010). Glucose intolerance in these patients is due to potentiated hepatic gluconeogenesis (Maratou et al., 2010). These effects might be related to the control that exerts THs in the expression of genes involved in glucose and lipid metabolism and suggests that several physiological aberrations are common to hyperthyroidism and T2DM, which contribute to the loss of metabolic homeostasis. Longitudinal studies have also investigated the association of alterations in thyroid function and the prevalence of DM and metabolic syndrome in older adults (Heima et al., 2013; Waring, et al., 2012). At baseline, individuals in the metabolic syndrome group exhibited significantly higher TSH values than individuals not included in the metabolic syndrome group. The authors indicated that increased circulating levels of TSH were associated with greater prevalence of metabolic syndrome, even in participants within the normal range (Waring et al., 2012). Another longitudinal study performed in Amsterdam also associated higher prevalence of metabolic syndrome and obesity with individuals exhibiting higher circulating levels of TSH (Heima et al., 2013). Separated research as also indicated that hypothyroidism is associated with insulin resistance and dyslipidemia (Dimitriadis et al., 2006; Gierach & Junik, 2015; Wang, 2013). Further evidence also indicates an increased risk of DM in patients with hypothyroidism and a systematic review reported the increased prevalence of even subclinical hypothyroidism in patients with T2DM (Gronich et al., 2015; Han et al., 2015). As opposed to compelling research indicating the association of DM and thyroid dysfunction, which is supported by the well‐described role of THs on glucose metabolism and insulin secretion, other studies have failed to link hypothyroidism to the development of T2DM (Ishay et al., 2009; Radaideh et al., 2004).

A growing evidence is associating alterations in thyroid function with other types of DM such as type 1 DM (T1DM) and gestational DM (GDM). Several studies have shown that patients with T1DM, an autoimmune disease, are prone to exhibit autoimmune thyroid diseases such as Hashimoto's thyroiditis and Graves’ disease. Current data indicate that up to 30% of adults with T1DM have thyroid diseases of autoimmune origin (Araujo et al., 2008; Shun et al., 2014). Genetic studies have revealed susceptibility genes for this syndrome, which include the human leukocyte antigen, cytotoxic T‐lymphocyte‐associated antigen 4, protein tyrosine phosphatase non‐receptor type 22, forkhead box P3, and the interleukin‐2 receptor alpha/CD25 gene region (Dittmar & Kahaly, 2010). These genes are involved in immunological synapse and T‐cell activation, suggesting that similar pathogenic processes occur in T1DM and thyroid diseases of autoimmune origin (Dittmar & Kahaly, 2010).

Gestational DM is a common complication that affects ~10% of all pregnancies associated with adverse pregnancy outcomes, such as preeclampsia, macrosomia, and caesarean delivery (International Association of Diabetes, 2010; Petra et al., 2019). Upon delivery, GDM disappears but in many cases different types of DM (GDM in a subsequent pregnancy or T2DM) can spur later in life (Martin et al., 1999; Seely & Solomon, 2003). Among the changes that occur during pregnancy, it is known that the placenta increases the secretion of pro‐inflammatory cytokines that induce insulin resistance to favor nutrient availability to the fetus (Kim et al., 2010). Under these circumstances (e.g., transient insulin resistance during pregnancy), GDM is the result of compromised capacity of pancreatic β‐cells to increase insulin secretion to compensate insulin resistance in insulin‐target tissues (Kuhl, 1991). Several reports have determined that maternal hypothyroidism predisposes the offspring to exhibit limited insulin secretion and to develop glucose intolerance, increasing the risk of T2DM in the offspring (Karbalaei et al., 2013). Moreover, separated reports have also determined that hypothyroidism is associated with GDM (Martin‐Montalvo et al., 2019; Sell et al., 2008). In this regard, we found several mutations in *PAX8* leading to hypothyroidism associated with the development of GDM, indicating that human GDM could have a genetic component (Martin‐Montalvo et al., 2019). Remarkably, this work has revealed that PAX8 expression in pancreatic islets modulates cellular pathways involved in cellular survival (Martin‐Montalvo et al., 2019).

#### 1.5.3 The physiological and pathophysiological role of thyroid hormones in the endocrine pancreas

One of the main organs involved in the control of circulating glucose levels is the endocrine pancreas. Extensive research has demonstrated the role of THs in the differentiation, maturation, and functionality of metabolic tissues (Figure 3) (Mastracci & Evans‐Molina, 2014). *In vivo* research has determined that during postnatal development circulating levels of T3 increase and induce the expression of the MAF bZIP transcription factor A (MAFA) and THRs in pancreatic β‐cells to facilitate their maturation (Aguayo‐Mazzucato et al., 2011, 2013, 2015). Experiments performed in adult wild‐type mice have also indicated severe effects of TH supplementation in β‐cells, enhancing concomitantly β‐cell proliferation and apoptosis (Lopez‐Noriega et al., 2017). Remarkably, β‐cells of mice treated with T4 exhibit increased glucokinase (GK) expression (Figure 3). Enhanced GK activity is associated with increased β‐cell proliferation and apoptosis, which might facilitate a rapid β‐cell turnover (Kassem et al., 2000, 2010; Lopez‐Noriega et al., 2017). THs are involved in β‐cell aging since they induce the expression of the senescence marker p16INK4A (also known as CDKN2A). The effects of THs via binding to the different THR produce the maturation (MAFA) and the senescence (p16INK4A) of β‐cells. THRβ1 binds to the TRE site 2 of *MAFA* promoter, and THRα binds to the TRE site 5 of the *CDKN2A* promoter (Aguayo‐Mazzucato et al., 2018). At the organismic level, mice treated with T4 exhibit greater insulin expression and secretion in pancreatic islets under fasting conditions, indicating that the insulin secretion machinery is constitutively active to facilitate nutrient uptake by insulin‐target tissues (Figure 3) (Lopez‐Noriega et al., 2017).

Separated research in mild hypothyroid PAX8 heterozygous knockout mice, which exhibit several hallmarks of T2DM, has indicated that pancreatic islets exhibit a transcriptional profile associated with increased metabolic activity and impaired antioxidant capacity (Lopez‐Noriega et al., 2019). Pancreatic β‐cells are particularly vulnerable to oxidative stress due to a very limited expression of antioxidant genes, such as catalase and glutathione peroxidase (e.g. less than 5% of hepatic levels) (Tiedge et al., 1997). More importantly, under typical situations of cellular stress such as high glucose, high oxygen, or heat shock, pancreatic islets have a virtually absent capacity to increase the expression of antioxidant enzymes (Tiedge et al., 1997). Situations of enhanced metabolic activity concomitant with restricted antioxidant defenses can generate oxidative stress, which may lead to the accumulation of oxidative damage. Under these situations, if cellular stress is not resolved pancreatic endocrine function might be compromised and apoptotic processes might be initiated (Supale et al., 2012; Tiedge et al., 1997).

#### 1.5.4 Thyroid hormone‐related alterations in insulin‐target tissues

Alterations in TH function have tremendous effects in liver tissue (Figure 3). THs induce increases in intracellular glucose production and insulin resistance (Klieverik et al., 2008). TH‐mediated insulin resistance might be produced by increased levels of cytokines generated in peripheral tissues, such as the adipose tissue (Gierach et al., 2014; Mitrou et al., 2010). Compromised insulin sensitivity produced by THs can per se have important consequences for glucose homeostasis given the central role of insulin action on the regulation of hepatic gluconeogenesis and glycogenolysis (Hatting et al., 2018). Interestingly, the effects on endogenous glucose production in the liver have been shown to be mediated, in some part, by the effects of THs in the paraventricular nucleus of the hypothalamus that mediate effects via sympathetic projections to the liver (Klieverik et al., 2008, 2009). In this regard, elegant studies conducted by Klieverik et al. have shown that the increases in endogenous glucose production mediated by the paraventricular nucleus are independent of circulating levels of glucoregulatory hormones (Klieverik et al., 2009; Martin et al., 1999). Moreover, these studies have demonstrated that hepatic sympathetic denervation entirely blunts the paraventricular TH‐induced increase in endogenous glucose production.

Non‐alcoholic fatty liver disease (NAFLD) is the hepatic manifestation of metabolic syndrome. Several alterations have been found in the pathogenesis of NAFLD in hypothyroid patients, which include the development of insulin resistance, dyslipidemia, and increased adiposity (Dimitriadis et al., 2006; Pagadala et al., 2012; Pucci et al., 2000; Waring, et al., 2012). Epidemiological studies have determined the existence of an inverse correlation between circulating TH levels and the incidence of NAFLD (Ludwig et al., 2015). Separated evidence has also indicated that patients with NAFLD exhibit higher serum TSH levels and lower free T4 levels (Xu et al., 2011). In this line, hypothyroidism is more frequent in patients with NAFLD when compared to healthy individuals matched for ethnicity, age, sex, and body mass index. Hypothyroidism was also higher in patients with non‐alcoholic steatohepatitis (NASH), a more serious form of fatty liver disease, when compared to patients suffering NAFLD without NASH. Individuals diagnosed with hypothyroidism were 2.1 (95% confidence interval: 1.1–3.9, *p* = 0.02) and 3.8 (95% confidence interval: 2–6.9, *p* < 0.001) times more likely to suffer NAFLD and NASH, respectively (Pagadala et al., 2012). Moreover, NASH and advanced fibrosis are more prevalent in patients with overt and subclinical hypothyroidism (Kim et al., 2018). Interestingly, besides overt and subclinical hypothyroidism, even upper TSH levels within the euthyroid range have also been associated with NAFLD, irrespective of well‐established metabolic risk factors (Bano et al., 2016; Chung et al., 2012; Pagadala et al., 2012). Moreover, hypothyroid patients exhibit increased esterification of hepatic fatty acids with restricted lipoprotein lipase activity and decreased hepatic uptake of HDL, indicating an improper cholesterol metabolism (Pearce, 2004; Pucci et al., 2000). Mild hypothyroid PAX8 heterozygous knockout mice exhibit increased hepatic levels of *CD36* expression (Lopez‐Noriega et al., 2019), a long‐chain fatty acid translocase that participates in fatty acid uptake (Greco et al., 2008; Pepino et al., 2014). Increased fatty acid uptake mediated by *CD36* might contribute to potentiate hepatic lipid accumulation in mild hypothyroid mice (Lopez‐Noriega et al., 2019).

THs play also a significant role in other insulin‐target tissues such as the adipose tissue and the skeletal muscle (Figure 3). The association of hypothyroidism and compromised insulin‐stimulated glucose uptake in muscle and adipose tissue has been documented on animals and humans (Dimitriadis et al., 2006; Pagadala et al., 2012; Rochon et al., 2003). Nonetheless, conflicting results have been obtained when hyperthyroid patients were analyzed for positron emission tomography with 2‐deoxy‐2‐[fluorine‐18]fluoro‐d‐glucose integrated with computed tomography (^18^F‐FDG PET/CT). One report indicated that hyperthyroid patients exhibit increased radioactive glucose uptake in brown adipose tissue (BAT) when compared to euthyroid patients (Lahesmaa et al., 2014). A second study obtained similar results (e.g., increased radioactive glucose uptake) in hypothyroid patients suffering thyroid carcinomas when these patients became mildly hyperthyroid upon TSH suppression (Broeders et al., 2016). However, other studies have shown no differences in glucose uptake in patients with hyperthyroidism (Zhang et al., 2014) and in hypothyroid patients with thyroid cancer rendered thyrotoxic (Gavrila et al., 2017).

Interestingly, the effects of THs on WAT browning have been described, indicating that T4 supplementation for 14 days produces a marked increase in radioactive glucose uptake in suprascapular subcutaneous WAT regions (Figure 3). Transcriptional analyses indicated a marked induction of UCP1 and DIO2 expression, which suggests increased energy expenditure via thermogenesis (Skarulis et al., 2010). Given the inconsistent results on BAT function in hyperthyroid subjects, it is plausible that the TH effects on WAT are more physiologically relevant than the effects on BAT. Supporting this hypothesis, research carried out in rats has indicated that triiodothyroacetic acid induces the expression of UCP1 in abdominal WAT (Medina‐Gomez et al., 2008) and long‐term administration of the THRβ analog sobetirome produced the browning of subcutaneous WAT in obese rodents (Lin et al., 2015; Villicev et al., 2007). Remarkably, sobetirome administration to ob/ob mice produced a decrease in BAT thermogenic function, further suggesting that metabolic effects of sobetirome are mediated by WAT browning. Interestingly, hypothyroidism is also associated with increased WAT browning (Weiner et al., 2016). Weiner et al. determined that WATs at different locations of hypothyroid mice exhibit markers of WAT browning, such as multilocular UCP1 expression and reduced BAT activity using (Nunez et al., 2008) F‐FDG PET/CT determinations (Weiner et al., 2016). These data indicate that under conditions of compromised BAT functionality compensatory WAT browning occurs.

THs exert important influences in energy metabolism, which predisposes tissues and organs with a high metabolic demand, such as skeletal muscle, to severe effects in patients suffering hypothyroidism and hyperthyroidism (Figure 3). In skeletal muscle, glucose uptake represents the limiting step in glucose metabolism and it is mediated by the plasma membrane glucose transporter 4 (Glut4). THs stimulate Glut4 expression through a positive TRE (DR+4) site in its promoter, which represents a direct link with carbohydrate metabolism (Torrance et al., 1997). Studies focused to determine the physiological and pathophysiological role of THs in skeletal muscle have indicated that up to 57% of patients with hypothyroidism exhibit muscle damage, revealed by high levels of creatine kinase (Hekimsoy & Oktem, 2005). Remarkably, TH treatment on hypothyroid patients reduced creatine kinase levels and improved muscle complications (Hekimsoy & Oktem, 2005; Rodolico et al., 1998). Individuals suffering hyperthyroidism also display muscle weakness, and the normalization of TH function in these patients increases muscle strength and muscle cross‐sectional area (Brennan et al., 2006). Insulin responsiveness is improved upon TH administration in patients and experimental models of hypothyroidism (Lopez‐Noriega et al., 2019; Rochon et al., 2003). At the tissue level, THs promote insulin sensitivity in skeletal muscle, an effect that depends on the proper conversion of T4 to T3 by DIO2. Accordingly, cell cultures of myotubes developed from DIO2 knockout mice and DIO2 knockout mice exhibit insulin resistance (Table 1) (Grozovsky et al., 2009; Marsili et al., 2011). *In vivo* research mice treated with T4 rendered hyperthyroid has shown that insulin signaling is chronically activated in skeletal muscle lysates, which might be detrimental at long term (Figure 3) (Lopez‐Noriega et al., 2017).

#### 1.5.5 Therapeutic approaches based on thyroid hormones or thyromimetics for diabetes mellitus

As previously mentioned hypothyroidism is associated with metabolic deregulations that increase the propensity to develop T2DM. TH supplementation normalizes parameters associated with DM, such as lipid and lipoprotein levels, diminishing the risk of developing this disease (Tzotzas et al., 2000). Studies in mice have also supported the potential of THs to improve metabolic health. TH supplementation has been shown to enhance glucose tolerance in wild‐type mice (Lopez‐Noriega et al., 2017) and to attenuate hyperglycemia in leptin receptor‐deficient mice (Lin & Sun, 2011). Remarkably, research in mice has also determined the potential of THs to improve metabolic health and survival in experimental models of T1DM (Lopez‐Noriega et al., 2017; Verga Falzacappa et al., 2009). Our results indicated that levothyroxine supplementation blunted the onset of experimental T1DM using the RIP‐B7.1 model, which recapitulates the β‐cell‐specific autoimmune attack that suffer patients with T1DM (Lopez‐Noriega et al., 2017). Interventional studies in humans using levothyroxine and the thyroxine enantiomer dextrothyroxine indicated that serum levels of LDL cholesterol were improved upon treatment. However, treatments were discontinued because participants developed serious adverse effects, substantiating the narrow therapeutic window of interventions based on the use of THs (Ochs et al., 2008; Sherman et al., 1997). Notwithstanding, the beneficial effects of interventions based on the use of THs in certain metabolic parameters have prompted the development of thyromimetics as promising agents to improve metabolic health. Newly generated thyromimetics could in principle bestow therapeutic benefits in specific cell types or organs producing an improvement of metabolic homeostasis, while avoiding side effects (Finan et al., 2016; Shoemaker et al., 2012). As such, fatty liver might be treated with thyromimetics designed to target specifically hepatic tissue (Taylor et al., 1997). An elegant work from Finan et al. (2016) has determined that the liver could be directly targeted using a glucagon‐T3 mixed agonist, which mediates the selective delivery of T3 to the liver. In principle, similar approaches could potentially be effective to target the WAT, BAT, or pancreatic islets, since THs are known to play an important role in these tissues (Gavrila et al., 2017; Lopez‐Noriega et al., 2017). Separated research has also investigated interventions targeting THRα1 or THRβ1 to obtain beneficial effects in target tissues while avoiding undesirable effects on other target organs (Mishra et al., 2010). The THRβ agonists sobetirome and eprotirome have proved effectiveness in several experimental models reducing circulating LDL levels without altering heart rate (Martagon et al., 2015; Perra et al., 2008; Sharma et al., 2014). Separated research has also shown beneficial effects in hepatic steatosis, cholesterol, and triglyceride levels in rodents (Cable et al., 2009; Erion et al., 2007; Grover et al., 2003). However, in several cases, thyromimetics (e.g., eprotirome and DIPTA) have failed due to inefficacy or toxicity in human or preclinical research (Ladenson et al., 2010; Sjouke et al., 2014; van der Valk. et al., 2014). Remarkably, the selective THRβ agonists MGL‐3196 and VK2809 have recently been investigated as lipid‐lowering agents and in phase 2 clinical trials to treat NASH (Lonardo et al., 2017; Sinha et al., 2019; Taub et al., 2013). The latest results using MGL‐3196 in humans indicate a significant reduction in hepatosteatosis after 12 and 36 weeks of treatment (Harrison et al., 2019; Sinha et al., 2019). Moreover, VK2809 has been shown to reduce hepatic lipid content and circulating LDL levels (Sinha et al., 2019). However, given the potential side effects of thyromimetics, a better understanding of their selective metabolic actions and experimental and clinical studies evaluating the long‐term effects of these interventions is needed. Altogether, we believe that the proof of concept of the efficacy of thyromimetics has been established in several pathophysiological contexts. Sufficient arguments are available to foster research to generate site‐specific modulators of TH function for DM and other diseases.

### 1.6 Thyroid hormones in cancer

#### 1.6.1 The alterations of thyroid hormone function and cancer

In the scientific literature, there are enough evidences to clearly state that TH dysfunction increases the risk to develop different types of cancers, as elegantly reviewed by Liu et al. (2019). Recent reports have indicated that hyperthyroidism is associated with higher risks to develop thyroid cancer, breast cancer, and prostate cancer, while hypothyroidism is associated with a higher risk to develop thyroid cancer specifically within the first 10 years of follow‐up (Hellevik et al., 2009; Kim et al., 2019; Tran et al., 2020). Another report has indicated that subclinical hypothyroid women without a previous history of thyroid disease have higher risk of breast cancer, bone cancer, or skin cancer (Tseng et al., 2015), whereas a different investigation indicates that breast cancer in hypothyroid women was associated with lower risk and better prognostic markers such as smaller tumor size or fewer metastases (Cristofanilli et al., 2005). Pertinent questions arise from epidemiological studies that in the majority of the cases have not been addressed. In this regard, information related to treatments used to normalize thyroid function, which might influence the risk, progression, and mortality of cancer, is not described (Hellevik et al., 2009; Kim et al., 2019; Pinter et al., 2017; Tseng et al., 2015). Limitations on clinical studies and conflicting results in the literature indicate that further studies are required to define the exact contribution of TH deregulations in carcinogenesis and cancer progression.

Of particular interest is the association of hepatocellular carcinoma (HCC) and hypothyroidism. Historically, the main known causes of HCC are viral hepatitis viruses, tobacco smoking, alcohol abuse, non‐alcoholic steatohepatitis, T1DM, T2DM, autoimmune hepatitis, aflatoxin, and cirrhosis (Adami et al., 1996; Bosetti et al., 2014; Dragani, 2010; Hassan et al., 2002). However, genetic predisposition might play a role in the risk of HCC since it has been estimated that ~1/5 of diagnosed HCCs in the United States of America are not associated with known predisposing risk factors (El‐Serag & Mason, 2000). Clinical findings have supported that hypothyroidism predisposes to HCC development, suggesting that improper TH function might represent a risk factor for this type of cancer (Hassan et al., 2009; Reddy et al., 2007). Reddy et al. reported a significantly increased odds ratio of 6.8 (95% confidence interval, 1.1–42.1) of hypothyroidism in patients with HCC with unknown cancer etiology when compared to patients with HCC with alcoholic liver disease or hepatitis C after adjusting for confounding factors. In this report, a twofold higher risk of HCC was determined for subjects with hypothyroidism when compared to patients without thyroid disorders. This association was specifically significant in female patients, even when analyses were adjusted to gender as a covariate. In this line, supportive research further analyzed the association of hypothyroidism and HCC risk in men and women (Hassan et al., 2009). In this work, authors determined that long‐term (10 years or more) hypothyroidism was associated with greater risk of HCC specifically in women. Remarkably, the association between hypothyroidism and HCC in women was independent of other established risk factors for HCC. Supporting the role of thyroid dysfunction in HCC progression, TSH levels greater than 3.77 mIU/L have been associated with larger HCCs and increased levels of free T4 at the time of diagnosis have been associated with reduced survival (Pinter et al., 2017). Separated research in mammals indicates that hypothyroidism can directly cause liver cell damage, representing a risk factor for spontaneous liver cancer. Indeed, PAX8 heterozygous knockout mice, which have a direct disorder in thyroid tissue leading to a mild hypothyroidism, have a ~ threefold increased incidence of liver cancers (Lopez‐Noriega et al., 2019). In principle, since hepatocarcinogenesis is a multistep process that originates from premalignant lesions, the severity of hypothyroidism and the effectiveness of treatments to normalize TH function might affect the risk to develop HCC.

#### 1.6.2 Molecular mechanisms of cancer in thyroid hormone dysfunction

The role of TH signaling in cancer has also been investigated, and growing evidences indicate that THRs play a significant role in inhibiting the proliferation, transformation, progression, invasion, and metastatic processes in tumors (Garcia‐Silva & Aranda, 2004; Garcia‐Silva et al., 2011; Liu et al., 2019). Supporting the role of THRs in cancer, the lack of expression or mutations on THRs has been identified in several tumors, such as HCC, renal cell carcinomas, and papillary thyroid carcinomas (Table 1) (Chan & Privalsky, 2006; Kamiya et al., 2002; Kim & Cheng, 1830; Lin et al., 1999; Puzianowska‐Kuznicka et al., 2002). Remarkably, several reports have described that ~70% of HCC harbor mutations in THRα or THRβ and several tumors exhibit mutation in both loci (Chan & Privalsky, 2006; Kim & Cheng, 1830; Lin et al., 1999). However, despite compelling evidence linking THR gene mutations to several cancers, other reports have not identified THR mutations in deep sequencing analysis of HCCs (Ahn et al., 2014; Cleary et al., 2013; Guichard et al., 2012; Schulze et al., 2015; Totoki et al., 2014), indicating that further research must be performed to fully determine the role of THR mutations in HCC. Notwithstanding, researchers have proposed that THRs act as tumor suppressors via inhibition of WNT signaling, inhibition of the expression of CDK2 and cyclin E, and stimulation of TGFβ signaling. These alterations lead to cell cycle arrest at the G1 phase of cell cycle (Yen et al., 2006). Mutant variants of TRHs, such as THRα‐V390A and THRα‐E350 K/P398S, act as dominant negative of the wild‐type THRs and might escape from these regulatory mechanisms, promoting cancer progression (Chan & Privalsky, 2006; Lin et al., 1997; Sinha et al., 2018). The main effects of these mutations are the impairment of T3 binding and altered recognition of TRE, leading to deregulation of the expression of THR targets (Rosen & Privalsky, 2011). In this regard, whereas wild‐type THRs modulate c‐Jun/AP‐1 function and suppress anchorage‐independent growth, THR mutants are ineffective (Chan & Privalsky, 2006). Research in animals has indicated that mice transgenic for *v*‐*erbA*, an avian retroviral gag gene fused to a mutated THR that produces a dominant negative of the THRα, exhibited hypothyroidism and increased incidence of HCCs in male mice (58% in transgenic mice vs. 8% in control mice) (Barlow et al., 1994). These data further indicate that inadequate TH signaling predisposes to liver cancer in male mice (Barlow et al., 1994). Reports have indicated that early in the tumorigenic process, there is a restriction on THRα1 and THRβ1 expression, which favors the progression to HCC (Frau et al., 2015). Interference of THRβ1 expression promoted cell growth and migration in HCC cells. Moreover, down‐regulation of THRβ1 induced the proliferative capacity, indicating that this receptor is a negative regulator of cell replication and that hypothyroidism favors the progression of HCC (Frau et al., 2015). These data suggest that THR agonists could be studied as therapeutic targets to block HCC development and progression.

Additional research has also pointed that other components of TH signaling might affect different aspects of carcinogenesis and cancer progression. In particular, the interaction of T4 with integrin αvβ3 has been shown to play a significant role in cancer progression and invasion. Integrin αvβ3 is a cell adhesion molecule that connects the cytoskeleton with the extracellular matrix or other cells. Interaction of T4 with integrin αvβ3 is a non‐genomic action of THs that supports angiogenesis. This process facilitates the generation of new blood vessels during development and wound healing (Al Husseini et al., 2013; Liu et al., 2014; Zhang et al., 2019). However, the same process favors cancer progression (Davis et al., 2016; Schmohl et al., 2019). At mechanistic level, T4‐activated integrin αvβ3 enhances signaling non‐genomic action of THs via activation of MAPK/ERK1/2 and/or PI3 K signaling. Activation of these signaling pathways produces cellular proliferation, which might favor cancer progression, and blockade of apoptotic processes and metastasis (Davis et al., 2016; Mousa et al., 2012, 2018; Weingarten et al., 2018). Moreover, separated evidences indicate that differences in deiodinase expression and differential splicing have been found in several cancers (Meyer et al., 2008) and increased TSHR expression have been detected in liver, ovarian, lung, and breast cancers (Table 1) (Govindaraj et al., 2012; Gyftaki et al., 2014; Kim et al., 2012; Shih et al., 2018; Tian et al., 2010). Moreover, it is known that TSHR is functional in the majority of human HCCs and that high TSHR expression is correlated with unfavorable postoperative outcome in patients with HCC receiving surgical treatment (Shih et al., 2018). These results suggest that high TSH levels might confer pathophysiological advantage to HCCs. Therefore, it is tempting to speculate that alterations in TH signaling due to changes in deiodinase or TSHR expression and/or activity could also play a role in tumor progression. Therefore, deiodinase or TSHR expression or activation could be studied as markers for cancer diagnosis (Piekielko‐Witkowska et al., 2009).

Alterations in thyroid function have been associated with mitochondrial dysfunction, leading to increased generation of mitochondrial reactive oxygen species (ROS) and altered antioxidant defenses such as superoxide dismutase, catalase, and glutathione (Grattagliano et al., 2003; Lopez‐Noriega et al., 2019; Venditti et al., 2003; Videla et al., 2015; Zhao et al., 2020). In this regard, an elegant study has recently shown that mitochondria isolated from the liver of hyperthyroid rats display an increase on oxygen consumption, the generation of hydrogen peroxide production, and the accumulation of lipid peroxidation, while present a reduced efficiency of oxidative phosphorylation (Venediktova et al., 2020). Remarkably, experiments using a model of hypothyroidism in rats have also shown that, in liver mitochondrial isolations, mitochondrial respiration is decreased and the capacity of mitochondria to remove hydrogen peroxide is compromised (Venditti et al., 2003). If antioxidant responses are not capable of restoring cellular homeostasis, increased ROS generation results in the accumulation of oxidative damage to macromolecules including lipids, proteins, and DNA (Mancini et al., 2016). ROS induce nicks in DNA that, if not repaired, contribute to premature senescence and carcinogenic processes on susceptible cells. T3 is known to increase the levels of 8‐oxo‐2'‐deoxyguanosine (8‐OH‐dG), a biomarker of oxidative DNA damage. Moreover, a strong cofocalization of 8‐OH‐dG with TP53BP1 has supported the presence of oxidized DNA at double‐strand break sites. Supporting the role of TH‐induced oxidative stress leading to DNA damage, antioxidant treatment using *N*‐acetyl‐l‐cysteine in TH‐treated samples blunted formation of 8‐OH‐dG/TP53BP1 foci (Zambrano et al., 2014). Several studies have pinpointed that the accumulation of liver damage in hypothyroidism and the increased propensity to develop HCC could be mediated by the accumulation of oxidative damage (Baskol et al., 2007; Lopez‐Noriega et al., 2019; Nanda et al., 2007). Mild hypothyroid PAX8 heterozygous knockout mice exhibit an ~threefold increase in the prevalence of liver cancers (Table 1). Analyses in liver tissues isolated before the initiation of cancer occurrence indicated the existence of increased ROS production and accumulation of oxidative damage, suggesting a causal link with carcinogenesis. Data generated in the murine model of PAX8 deficiency suggest that humans bearing *PAX8* mutations might have greater propensity to develop metabolic complications and liver cancer. In this line, a study investigated the associations of two single nucleotide polymorphisms (SNPs) on *PAX8* with HCC survival (Table 1) (Ma et al., 2017). In this report, patients harboring the rs1110839 GT/GG genotypes and rs4848320 CT/TT genotypes exhibited longer survival time than patients harboring the rs1110839 TT and rs4848320 CC genotypes. Moreover, multivariable Cox regression analysis showed that these SNPs had a significant prognostic value for HCC, suggesting that *PAX8* gene might be considered a biomarker to predict HCC survival.

Interestingly, the scientific literature indicates that, despite the known pro‐proliferative actions of THs, potentiated cellular proliferation driven by THs is not sufficient *per se* to enhance HCC progression (Columbano et al., 2006, 2008; Ledda‐Columbano et al., 2000; Perra et al., 2009). An elegant report investigating THR knockout mice has supported that TH function has a dual role in tumor development (Martinez‐Iglesias, Garcia‐Silva, Tenbaum, et al., 2009). Research performed in THRα‐THRβ double homozygous knockout mice showed that the lack of THR results in the restriction of benign tumor formation at early stages of skin carcinogenesis, but enhanced malignant transformation at the later stages of the disease. These mice developed fewer number of tumors than wild‐type mice (Martinez‐Iglesias, Garcia‐Silva, Tenbaum, et al., 2009). Subsequent work on experimental models of cancer using nude mice indicated that systemic hypothyroidism slows tumor growth but potentiates metastatic processes in a process that is independent of THRβ1 expression (Martinez‐Iglesias, Garcia‐Siva, Regadera, et al., 2009). Hypothyroid mice exhibited greater number of spontaneous metastasis in tissues such as lung, liver, or bone, when compared to euthyroid hosts. These effects were determined in hepatoma and breast cancer cells that stably express THRβ1, indicating that systemic TH function is more relevant than direct effects of THRβ1 on tumorigenesis (Martinez‐Iglesias, Garcia‐Siva, Regadera, et al., 2009). These results support the notion that restricted thyroid function favors cancer metastasis.

#### 1.6.3 Therapeutic approaches based on thyroid hormones or thyromimetics for cancer

In the late nineteenth century, Beatson proposed the use of thyroid extract in conjunction with oophorectomy as a therapy for breast cancer (Beatson, 1896). In this line, TH treatment has been shown to reduce HCC progression, development of lung metastases, and the risk of colorectal cancer (Frau et al., 2015; Kowalik et al., 2020; Ledda‐Columbano et al., 2000; Rennert et al., 2010). Despite the fact that THs are known to induce hepatocyte proliferation, TH administration or treatments with the THRβ agonist sobetirome induces regression of carcinogen‐induced hepatic nodules in xenobiotic‐based research models (Perra et al., 2009). These findings support the exploration of treatments based on the use of THs or thyromimetics for cancer treatment. However, it is unlikely that current thyromimetics, such as sobetirome, could be used in the clinical practice for cancer treatment given their potential severe side effects (Lammel Lindemann & Webb, 2016). Moreover, separated evidences indicate that TH treatments for hypothyroidism are associated with increased risk of renal carcinomas and breast cancer (Kapdi & Wolfe, 1976; Rosenberg et al., 1990), suggesting that TH supplementation might accelerate tumor growth or recurrence (Hercbergs, 1996, 1999). Notwithstanding, the use of tissue‐specific thyromimetics devoid of toxicity might be realistic in the future for cancer treatment, but further studies are required to determine their safety and efficacy.

### 1.7 Concluding remarks

THs are essential hormones that orchestrate whole‐body metabolism acting on every single cell of the body. Alterations in TH function have been associated with both disease and health, ranging from exceptional long longevity in individuals with a low thyroid function to the lack of viability in individuals devoid of THs. Studies in humans have demonstrated that the oldest old exhibit restricted TH function. However, studies directly targeting TH production do not extend life span, indicating that a genetic or epigenetic signature is required to achieve the longest life expectancy. Alterations in THs leading to different forms of hyperthyroidism and hypothyroidism have been associated with several age‐related diseases, such as DM and cancer. In this regard, different types of DM have been determined as risk factors to develop certain types of cancer. The association of TH alterations with different aspects of the pathogenesis of DM and cancer and overall life expectancy suggests that aging, DM, and cancer share certain mechanisms of pathogenesis in which THs contribute. Therefore, it is tempting to speculate that treatments or interventions designed to treat DM might have positive effects on cancer and *vice versa*, which might also have beneficial consequences on aging and life expectancy. Further research is required to increase knowledge on the alterations that occur in early stages of DM and cancer related to THs, which currently limits the identification of potential biomarkers and targets to facilitate the diagnosis, the prognosis, and the development of novel therapies for DM and cancer. The pleiotropic effects of TH alterations in different tissues indicate that personalized and precision medicine must be applied to provide the optimal treatment for each patient. The narrow therapeutic window of interventions based on the use of natural THs has prompted the development of thyromimetics. However, the majority of thyromimetics tested to date have failed due to inefficacy or toxicity. Notwithstanding, the latest research indicates that there is hope in the development of safe and effective thyromimetics that might provide therapeutic benefit for different diseases including cancer and DM.

## CONFLICT OF INTEREST

The authors declare no conflict of interest.

## AUTHOR CONTRIBUTIONS

A.M‐M conceived the manuscript. All authors contributed to the manuscript revision and approved the submitted version.
